# Nanoscale momentum-resolved vibrational spectroscopy

**DOI:** 10.1126/sciadv.aar7495

**Published:** 2018-06-15

**Authors:** Fredrik S. Hage, Rebecca J. Nicholls, Jonathan R. Yates, Dougal G. McCulloch, Tracy C. Lovejoy, Niklas Dellby, Ondrej L. Krivanek, Keith Refson, Quentin M. Ramasse

**Affiliations:** 1 SuperSTEM Laboratory, SciTech Daresbury Campus, Keckwick Lane, Daresbury WA4 4AD, UK.; 2 Department of Materials, University of Oxford, Parks Road, Oxford OX1 3PH, UK.; 3 Physics, School of Science, RMIT University, Melbourne, Victoria 3001, Australia.; 4 Nion Company, 11511 NE 118th Street, Kirkland, WA 98034, USA.; 5 Department of Physics, Arizona State University, Tempe, AZ 85287, USA.; 6 STFC (Science & Technology Facilities Council) Rutherford Appleton Laboratory, Harwell Science and Innovation Campus, Didcot OX11 0QX, UK.; 7 Department of Physics, Royal Holloway, University of London, Egham TW20 0EX, UK.; 8 School of Physics, University of Leeds, Leeds LS2 9JT, UK.; 9 School of Chemical and Process Engineering, University of Leeds, Leeds LS2 9JT, UK.

## Abstract

Vibrational modes affect fundamental physical properties such as the conduction of sound and heat and can be sensitive to nano- and atomic-scale structure. Probing the momentum transfer dependence of vibrational modes provides a wealth of information about a materials system; however, experimental work has been limited to essentially bulk and averaged surface approaches or to small wave vectors. We demonstrate a combined experimental and theoretical methodology for nanoscale mapping of optical and acoustic phonons across the first Brillouin zone, in the electron microscope, probing a volume ~10^10^ to 10^20^ times smaller than that of comparable bulk and surface techniques. In combination with more conventional electron microscopy techniques, the presented methodology should allow for direct correlation of nanoscale vibrational mode dispersions with atomic-scale structure and chemistry.

## Teaser

A widely applicable method for accessing phonon dispersions of materials at high spatial resolution is demonstrated.

## INTRODUCTION

Acoustic and optical phonons affect fundamental physical properties such as the conduction of sound and heat. For two-dimensional (2D) materials (*1*, *2*), van der Waals heterostructure devices (*3*), topological insulators (*4*, *5*), and thermoelectric materials (*6*–*8*), as well as many other materials systems relevant to fields spanning catalysis, biomedical, condensed matter physics, and chemistry, vibrational properties can depend on nano- and atomic-scale structure. However, until now, techniques available to probe the momentum transfer (*ℏ***q**) dependence or dispersion of these vibrational modes have been limited to essentially bulk approaches, such as inelastic neutron and x-ray scattering spectroscopies (*9*, *10*), reflection electron energy loss spectroscopy (REELS) measuring the average surface response (*11*, *12*), or optical techniques limited to **q** ~ 0 (where **q** is the wave vector) (*13*–*17*).

Here, we demonstrate a widely applicable experimental and theoretical methodology for nanoscale mapping of optical and acoustic phonons across the first Brillouin zone using electron energy loss spectroscopy (EELS) in the scanning transmission electron microscope (STEM), with unprecedented spatial resolution (*18*) down to <2 nm, probing a volume ~10^10^ to 10^20^ times smaller than previously done using inelastic neutron and x-ray scattering spectroscopies. Despite the unavoidable trade-off between the simultaneously achievable spatial and momentum resolution, our methodology carefully balances the experimental parameters to uncover a wealth of information, inaccessible through any other technique.

Our ab initio calculations show remarkable agreement with experiments. We assign unambiguously distinct features in hexagonal (h) and cubic (c) boron nitride (BN) EEL spectra to acoustic and optical (hBN and cBN) and in- and out-of-plane (hBN) phonon modes. This goes beyond recent field-changing proof-of-principle demonstrations of vibrational spectroscopy in the electron microscope (*18*–*22*) and opens the door to correlating **q**-dependent vibrational properties in nanomaterials directly with atomic-scale structure and chemistry, which is also accessible with STEM-EELS (*23*).

## RESULTS

### A combined experimental and theoretical approach to momentum-resolved vibrational spectroscopy

**Fig. 1:**
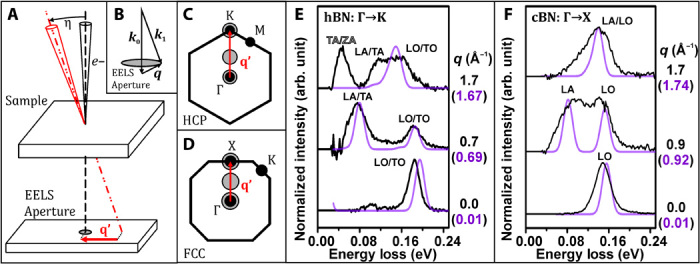
Sketch of the experimental scattering geometry and selected momentum-resolved spectra. (**A**) By tilting the incident beam by an angle η, the forward scattered beam is effectively displaced with respect to the spectrometer entrance aperture (in momentum space) by a distance and direction given by a wave vector **q′**. (**B**) **q** as defined by the incident (***k*_0_**) and scattered (***k*_1_**) wave vectors, for **q′**→0. Sketch of the first Brillouin zone of (**C**) a 2D-projected hexagonal close-packed (HCP) crystal and (**D**) a 2D-projected face-centered cubic (FCC) crystal illustrating how effectively displacing the EELS entrance aperture (gray discs) by **q′** with respect to the forward scattered beam results in EEL spectrum momentum selectivity. Selected momentum-resolved experimental (black) and simulated (in-plane polarization only, purple) EEL spectra for (**E**) hBN along Γ→K and (**F**) cBN along Γ→X. The magnitude of displacement in momentum space considered for each spectrum is given in black (experimental spectra, corresponding to the center of the displaced beam, estimated to one decimal place) and purple (modeled spectra).

Analogous to graphite and diamond, respectively, the hexagonal and cubic polymorphs of boron nitride are ideal model systems for the present study; the difference in crystallographic and electronic structure of sp^2^-bonded hBN and sp^3^-bonded cBN allows for an in-depth evaluation of both experimental and theoretical methodologies on nanoscale flakes of material. Figure 1 (A to D) illustrates how vibrational EEL spectra were resolved in momentum space in the present work, while maintaining a nanometer-sized electron probe, building on prior experimental approaches used for momentum-resolved core (*24*) and valence (*25*–*28*) EELS in the (S)TEM. The incident electron beam was tilted by an angle η, the magnitude of which determines the effective displacement wave vector (**q′**) of the spectrometer entrance aperture with respect to the forward scattered direction. Figure 1B illustrates the post-specimen scattering geometry for **q′** = 0 (that is, the circular spectrometer entrance aperture is centered on **q**→0). The assignment of loss peaks to specific phonon modes was made on the basis of ab initio modeling of mode energies and of calculations of the contribution of individual modes to the loss function; that is, the intensity of each mode in the EEL spectrum using a scattering function formalism. As with similar formalisms for x-rays and neutron scattering, this approach predicts that only phonon modes with a component along the direction of imparted momentum transfer will contribute to the EEL phonon spectrum (for more details, see Materials and Methods).

**Fig. 2:**
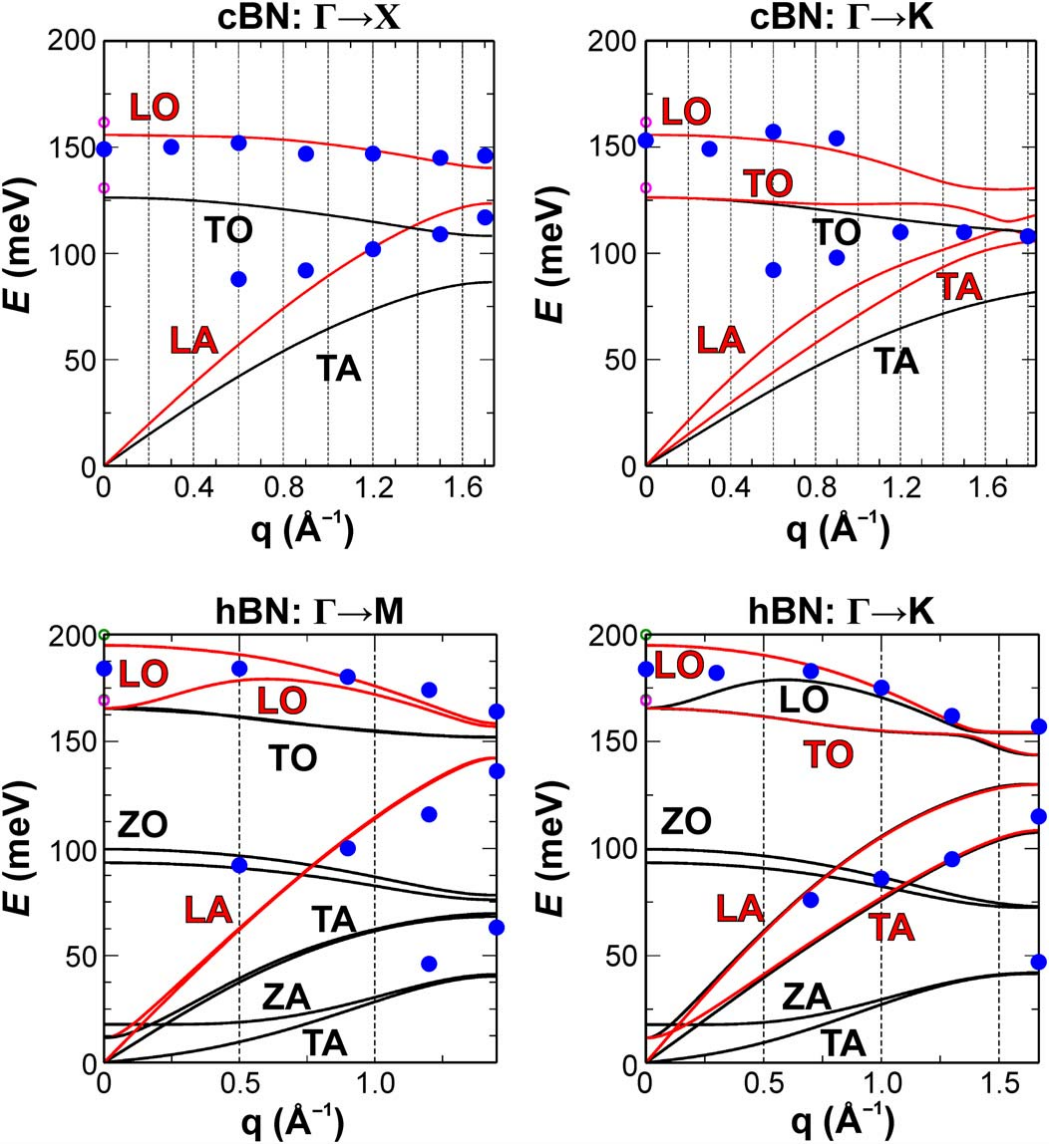
Simulated (full curves) and experimental (blue discs) hBN and cBN phonon dispersions. For in-plane polarization, only colored (red) modes are predicted to contribute to the EEL spectrum. Reported experimental Raman (pink circles) and infrared (green circles) values (*16*) (observed at **q**→0 only, showing the limitation of these optical techniques) are indicated.

Figure 1 (E and F) shows superimposed selected background subtracted experimental EEL spectra and simulated loss functions for hBN and cBN acquired from selected regions of their respective Brillouin zones along the Γ→K and Γ→X directions, indicated by the gray discs in Fig. 1 (C and D). The momentum resolution achieved in these specific experimental conditions was estimated to be Δ**q** = ±0.5 Å^−1^ (see Materials and Methods for a discussion of the parameters affecting the balance between spatial and momentum resolutions). For completeness, non–background-subtracted versions of the spectra in Fig. 1 (E and F) are also shown in fig. S1. Figure 2 shows the experimental peak values superimposed on the simulated phonon dispersions for hBN and cBN along the Γ→M/K and Γ→X/K directions, respectively, in good agreement with literature reports on bulk BN (*9*, *18*, *29*, *30*). Phonon branches in Fig. 2 are referred to by their behavior near the zone center. In the case of hBN, optical (O) and acoustic (A) modes are transverse (TO and TA), longitudinal (LO and LA), or out of plane (ZO and ZA).

### Interpreting the phonon mode contributions

The contributions of acoustic and optical modes are identifiable in the spectra in Fig. 1 (E and F) and follow the expected mode dispersions in Fig. 2. The most easily interpretable result is that of cBN in the Γ→X direction of its Brillouin zone (Figs. 1F and 2), where the two loss peaks can be attributed directly to either an individual LO or LA mode. Because of the 18- to 40-meV experimental energy resolution (see Materials and Methods), some modes could not be directly separated in the experimental spectra (see Figs. 1E and 2). This makes the interpretation of some spectra somewhat more complex, meaning the affected loss peaks are attributed to a combination of vibrational modes rather than to a single one. This in turn affects the match with calculated dispersion curves and may explain in particular the apparently reduced experimental slope of some mode dispersions compared to theory (Fig. 2). Furthermore, for a detailed comparison between theory and experiment, we need to consider the effect of experimental geometry. The experimental EEL spectrum can be understood as the integral of the double differential scattering cross section over all wave vectors admitted by the circular spectrometer collection aperture. The double differential scattering cross section is in turn proportional to the energy loss function multiplied by a (**q**-dependent) Lorentzian prefactor (*23*, *28*). In other words, the experimental geometry results in spectral “smearing” in **q**-space (that is, integration over a small range of wave vectors), while the nanometer-sized electron beam may probe local spatial inhomogeneities. Neither of these effects is currently taken into account in our theoretical approach. Thus, some of the discrepancies between the experimental data and the theoretical predictions, including the mismatch in experimental slope of some mode dispersions in Fig. 2, the larger than predicted LA/TA mode contribution at the K point of hBN in Fig. 1E, and the hBN LA/TA and cBN LA peak asymmetries at intermediate **q** in Fig. 1 (E and F), may be mitigated by including the effects of finite momentum integration and finite probe size in future calculations.

### Phonon polaritons and finite sample thickness

Interpreting the hBN “**q** ~ 0 Å^−1^” spectrum in Fig. 1E requires that the so-called *E*_1u_ LO-TO mode splitting (*18*, *29*) be taken into consideration. In “bulk” hBN, for small wave vectors near Γ, long-range electric fields associated with the E_1u_ LO mode result in a mode blueshift by about 30 meV with respect to the *E*_1u_ TO mode. At finite thicknesses, a range of modes with different energies appear between the LO and TO end members (that is, in the “upper Reststrahlen band”) (*13*, *15*, *29*). These modes are often referred to as phonon polaritons (PhPs), as they are attributed to coupling of optical phonons with photons in polar materials (*13*, *15*). Using infrared irradiation, Dai *et al*. (*13*) and, later, Shi *et al*. (*15*) showed experimentally (also confirming their results by theoretical calculations) that hBN PhP modes exhibit a significant thickness dependence. For hBN flakes with thicknesses of several hundred nanometers to a few atomic layers, PhP mode energies and wavelengths decrease with decreasing flake thickness, consistent with the earlier predictions in the study of Michel and Verberck (*29*). Batson and Lagos (*22*) recently showed that PhPs contribute significantly to the vibrational STEM-EEL spectrum of a thin hBN flake, demonstrating a 14-meV split between loss peaks attributed to the E_1u_ LO mode and PhPs, respectively. As typical STEM-EELS samples (including those used in the present work) have thicknesses of ≤~150 nm, the hBN LO/TO peaks in vibrational EEL spectra would thus be expected to show significant thickness dependence. Govyadinov *et al*. (*31*) recently showed in detail how the PhP thickness dependence significantly affects the hBN vibrational STEM-EEL LO/TO peak for small wave vectors. This effect is demonstrated in fig. S2 (see the Supplementary Materials), where the measured hBN LO/TO peak width increases and its maximum shifts to a higher energy loss as the nanometer-sized electron probe is moved into thicker regions of the hBN particle. In addition to the PhP thickness dependence, finite integration over **q** also contributes to the LO/TO peak energy shift and broadening. Thus, in vacuum [in “aloof” mode where the **q**→0 contribution dominates, that is, in a geometry probing primarily the PhP response (*31*)], the observed peak should be at its narrowest and its energy at the lowest, as we observe in our experiments (fig. S2), in agreement with the studies of Govyadinov *et al*. (*31*) and Nicholls *et al*. (*32*). While a “bulk crystal” model was used for our ab initio calculations, the present hBN experimental spectra were acquired from an electron transparent particle (that is, of finite thickness). Thus, in light of the above discussion, the difference in relative experimental and theoretical **q** ~ 0 Å^−1^ hBN LO/TO peak values must be understood as primarily due to a combination of PhP finite size effects and experimental geometry. As the Reststrahlen band of infrared spectra of cBN films also shows significant thickness dependence (*17*), we likewise attribute to the combination of PhP finite size effects and experimental geometry the minor discrepancy observed between the theoretical and experimental cBN LO **q** ~ 0 Å^−1^ peak in Fig. 1F.

### Balancing spatial and momentum resolution

Although phonon dispersion in BN has been extensively studied in the literature, including in the work of Rokuta *et al*. (*11*), who used high-resolution REELS to measure the average phonon dispersion of a monolayer hBN on a Ni(111) surface, the present methodology allows, in principle, for probing phonon dispersions of free-standing particles and features at the nanoscale. Here, the formation of an electron probe several orders of magnitude smaller than that used in REELS provides access to individual defects or nanoscale features and their possible influence on the phonon dispersion. Moreover, the STEM-EELS geometry uses a free-standing sample, which minimizes (and possibly avoids altogether) substrate effects, which have been reported to affect the measured vibrational REEL spectrum (*9*). The convergent beam geometry used in this work necessarily results in a modest momentum resolution compared to bulk optical and surface EELS techniques. Nevertheless, by carefully balancing the experimental parameters, a wealth of information about the vibrational response can be obtained, and our results show good agreement (taking into account the trade-off between momentum and spatial resolutions) with literature results (*9*, *11*, *18*, *29*, *30*).

### Out-of-plane mode contributions

**Fig. 3:**
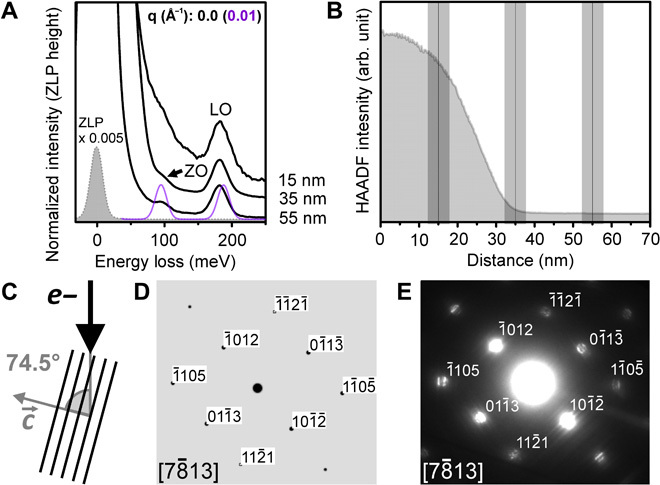
Spatially resolved vibrational EELS of hBN for an electron beam incidence of 74.5° to the crystallographic *c* axis. (**A**) Spatially resolved loss spectra acquired from a hBN particle in the regions indicated on the HAADF line profile in (**B**). A simulated spectrum (purple) is superimposed on the experimental aloof spectrum (distance, 55 nm). (**C**) Sketch showing the relative orientation of the incoming electron beam with respect to the hBN planes. The direction of the crystallographic *c* axis is indicated. (**D** and **E**) Simulated and experimental diffraction patterns of the hBN particle.

The modeled hBN spectra in Fig. 1E assume purely in-plane polarization. This is a good approximation for electron beam incidence parallel to the crystallographic *c* axis (Figs. 1E and 2) and small wave vectors. However, due to a combination of experimental geometry and Ewald sphere curvature, the effective contribution to the experimental spectrum of modes requiring out-of-plane polarization will increase with increasing momentum transfer. This trend is reflected in the present experimental data. For small wave vectors, the hBN out-of-plane mode contribution is negligible for electron beam incidence parallel to the crystallographic *c* axis (Figs. 1E and 2); however, Fig. 3 shows that a 74.5° angle between the incident electron beam direction and the crystallographic *c* axis results in a significant spectral contribution of the ZO mode and lower-energy ZA/TA modes. While ZA/TA modes contribute significantly to the spectrum for penetrating beam geometries, in aloof geometries, the acoustic mode contribution is minimized; thus, the ZO [and associated PhP (*15*)] mode contribution is observable as a separate peak, in good agreement with our modeled spectrum (Fig. 3A, black arrow). For large wave vectors (near and at the hBN K and M points), the experimental spectra exhibit low-energy peaks that can only be attributed to out-of-plane polarization of low-energy ZA/TA modes (Figs. 1E and 2). Hence, it is of great importance to carefully consider the effect of experimental geometry and Ewald sphere curvature when interpreting momentum-resolved vibrational STEM-EEL spectra. In addition to the effects discussed above, defects that break the symmetry of the crystal could also result in the nonzero contribution of out-of-plane modes.

## DISCUSSION

We have acquired momentum-resolved vibrational STEM-EEL spectra of hBN and cBN across the entire first Brillouin zone, using a ~1 nm–sized electron probe. We show that acoustic, optical, and anisotropic vibrational mode contributions are identifiable in the experimental data. Acoustic mode detection in particular is of crucial importance for real-life applications of vibrational spectroscopy in the electron microscope. The methodology outlined and demonstrated in the present work provides information akin to REELS (*11*, *12*), triple-axis neutron, and inelastic x-ray scattering spectroscopies (*9*, *10*), but with a much higher spatial resolution: using an electron probe size several orders of magnitude smaller than in REELS and sampling volumes 10^10^ to 10^20^ times smaller than x-ray and neutron spectroscopies. The present methodology provides information complementary to real space mapping of vibrational modes using tip-enhanced Raman imaging (*14*), infrared nanospectral imaging (*13*, *15*), or STEM-EELS (*18*, *21*). The energy resolution in STEM-EELS is presently poorer than that of the complementary techniques, but this is likely to improve in the future to around 5 meV (*33*). Because of the highly flexible optics of modern electron microscopes, the (necessarily modest) momentum resolution can be significantly increased at the cost of limiting the achievable spatial resolution, if required by the specific experiment. Conversely, increasing the spatial resolution to probe the effect of local inhomogeneities in the material on the energy loss response as a function of momentum, effectively exploring the switch-over between the local and nonlocal limits, could provide further fascinating insights into the scattering process. The ability to trade off spatial and momentum resolution within the same instrument is a unique feature of this experimental approach. Furthermore, combining the present approach with other more established STEM-EELS techniques (*23*) provides for a highly comprehensive atomic-scale structural and chemical characterization of material systems such as topological insulators (*4*, *5*), thermoelectric materials (*6*–*8*), 2D materials (*1*, *2*), and van der Waals heterostructure devices (*3*), where the vibrational response depends on nano- to atomic-scale structure and chemistry. As a particularly relevant example, in thermoelectric materials, the interplay between acoustic and anharmonic, so called rattler, vibrational modes is thought to play an essential role in controlling thermal conductivity (*6*). As rattlers are thought to be localized around specific structural features, the ability to directly probe these modes at the nanoscale in both real and momentum space should open a tantalizing new window onto the physics of these phenomena (*7*, *8*). Finally, we also expect the experimental methodology developed here to be applicable far beyond vibrational modes. As the achievable energy resolution improves with instrumentation developments, it should allow for nanoscale measurements of the dispersion of a wide range of low-energy excitations, including trions (*34*), Luttinger liquid (*35*) and terahertz charge carrier plasmons (*36*), and Higgs and Leggett modes in superconductors (*37*), among many others.

## MATERIALS AND METHODS

### Boron nitride

Samples investigated in the present work were fabricated from a commercially available hBN powder (*38*) and from a cBN powder synthesized under high-temperature and high-pressure conditions that was obtained from Van Moppes. The hBN and cBN powders were suspended in ethanol, sonicated, and dispersed onto separate standard 3-mm holey carbon TEM grids. Before insertion into the microscope, both samples were heated ex situ at 135°C for 6 hours in a vacuum (<1 × 10^−5^ Torr) to prevent adventitious carbon contamination during the experiments.

### STEM-EELS

Experiments were carried out at an acceleration voltage of 60 kV using a Nion UltraSTEM 100MC microscope (*39*) equipped with a Gatan Enfinium EEL ERS spectrometer optimized with high-stability electronics, installed at the SuperSTEM Laboratory, Daresbury, UK. In the (S)TEM, momentum-resolved EELS has previously been used to measure core [see, for example, (*24*) and references therein] and valence losses [see, for example, (*25*–*28*) and references therein]. Momentum-resolved EEL spectra can be acquired serially [see, for example, (*26*, *27*)] or in parallel [see, for example, (*24*, *25*, *28*)]. Here, spectra were acquired serially to optimize spectral signal-to-noise for all measured momentum transfers and to be able to probe the energy loss response along any direction in momentum space electron optically; that is, without the need for a dedicated tilt-rotation sample holder, which would possibly decrease mechanical stability between measurements along different high-symmetry directions of the Brillouin zone. In practice, EEL spectrum momentum selectivity was achieved by using the electromagnetic scan coils of the microscope to tilt the electron beam in increments corresponding to the desired magnitudes and directions of effective spectrometer entrance aperture displacement (**q′**) (see Fig. 1). For each momentum-resolved EEL spectrum, the magnitude and direction of **q′** were calibrated from the diffraction pattern formed by scanning the electron beam over the STEM bright-field detector as a function of beam tilt angle. The results presented in Figs. 1 (E and F) and 2 show that tilting the beam, either before (hBN measurements) or after (cBN measurements) the sample, results in momentum-resolved EEL spectra, where optical and acoustic mode contributions can be distinguished (see Fig. 1, E and F). In Figs. 1 (E and F) and 2, **q** values correspond to absolute values of wave vectors along the hBN Γ→K/M and cBN Γ→X/M directions of their respective Brillouin zones. Experimental **q** values are given by the magnitude of the effective spectrometer entrance aperture displacement along the hBN Γ→K/M and cBN Γ→X/M directions.

For the EELS measurements presented in Figs. 1 (E and F) and 2 and fig. S1, the electron beam collection semiangle (α) and spectrometer collection semiangle (β) were α = β ≈ 3 mrad. This experimental geometry was chosen to maintain a diffraction-limited probe size of ~1 nm while optimizing the spectral signal intensity, effectively limiting the momentum resolution to Δ**q** = ±0.5 Å^−1^. The momentum resolution was estimated from the effective collection semiangle β* = (α^2^ + β^2^)^0.5^ [see, for example, the book by Egerton (*23*)]. Because of the intrinsic trade-off between the simultaneously achievable momentum resolution and electron probe size, our particular experimental geometry necessarily results in a modest momentum resolution by comparison to optical techniques. However, we show that despite this, and owing to a carefully designed balance of experimental parameters, a wealth of information about the vibrational response of a material can be extracted that would not be available through any other technique. Furthermore, because of the highly flexible optics of the electron microscope, the momentum resolution can be significantly increased at the cost of limiting the achievable spatial resolution, if required. This ability and flexibility to balance momentum and spatial resolution as required are the keys to the methodology we describe. The energy resolution, measured as the full-width at half-maximum (Δ*E*) of the quasi-elastic zero loss peak (ZLP), decreased from 18–20 meV to 30–40 meV, with increasing momentum transfer. Because of the ~1/**q**^2^ vibrational EEL cross-section dependence, momentum-resolved spectra were acquired by summation over multiple acquisitions of 0.1 to 90 s (increasing with increasing **q′**), with a spectral sampling of 2 meV per channel. The increase in Δ*E* with increasing **q′** is thus likely due to ZLP energy drift increase with spectral acquisition time, in combination with a widening due to electron-atom Compton scattering (*40*) and, to a lesser extent, to electron optical aberrations in the post-specimen optics when using post-specimen beam tilt. Experimental peak values in Fig. 2 were determined by subtracting the ZLP tail of the corresponding raw EEL spectra (by fitting of a power-law function) and subsequently fitting the resulting spectrum with the minimum number of Gaussian functions deemed appropriate from the experimental peak shapes. In the context of momentum-resolved neutron scattering experiments, complex procedures have been developed to estimate a 3D resolution ellipsoid, describing the variations in momentum and energy resolutions as a function of scattering angle and energy loss (*41*). Here, the energy resolution of the spectrometer is, to an excellent approximation, constant as a function of scattering angle and mostly affected by instabilities that result in a broadening during long acquisitions at higher momentum transfer. Similarly, the momentum resolution is solely limited by the convergence and collection angles chosen in the present experimental conditions, so the more complex approach for determining a full-resolution ellipsoid used in neutron scattering was not deployed here.

Spectra in Fig. 3 were acquired with α = β ≈ 3 mrad, **q′** = 0, adding multiple spectra along the direction of an EEL spectrum image line scan, where each individual spectrum was acquired with an acquisition time of 0.1 s and a spectral sampling of 2 meV per channel. Spectra were acquired in both a penetrating (distance, <≈35 nm) and aloof geometry (distance, >≈35 nm). The spatial extent of spectral integration perpendicular to the particle “edge” (distance ≈ 35 nm) is indicated on the high-angle annular dark-field (HAADF) line profile in Fig. 3B. The experimental electron diffraction pattern in Fig. 3E was acquired with α ≈ 3 mrad. The simulated kinematic electron diffraction pattern in Fig. 3D was calculated using the SingleCrystal software by CrystalMaker Software Ltd.

For the EELS data presented in fig. S2, α = 31 mrad, β = 44 mrad, and **q′** = 0, the acquisition time was 5 ms, and the spectral sampling was 1 meV per channel. Several spectra were integrated over an EEL spectrum image to produce the spectra shown in Fig. 1A. Spectra were acquired in both a penetrating (distance, <≈73 nm) and aloof (distance, >≈73 nm) geometry. The spatial extent of spectral integration in the direction perpendicular to the particle edge (distance, ≈73 nm) is indicated on the HAADF line profile in fig. S2D. The spatial extent of spectral integration in the direction parallel to the particle edge was 31 nm for all spectra. For LO/TO measurements in fig. S2 (B, C, and E), the ZLP tail was subtracted by fitting of a power-law function, and each LO/TO peak was fitted with a Gaussian. The measured ZLP full-width at half-maximum is given as a function of electron beam position in fig. S2F.

The HAADF detector semiangles used for acquiring the line profiles in Fig. 3B and fig. S2D were 82 to 195 mrad. No postacquisition de-noising or deconvolution routine was used for any experimental spectrum.

### Ab initio modeling

Phonon dispersions for both hBN and cBN were calculated using first-order perturbation theory implemented in CASTEP (*42*, *43*), a pseudopotential density functional theory code. The contributions of the different modes to the loss function were then calculated using an approach based on a scattering function formalism, which shows that the double differential cross section for these vibrational modes depends on a scattering function at an energy *ℏ*ω and wave vector **q**. The scattering function contains a dot product between **q** and the phonon eigenvector, which, in agreement with other recent studies (*44*, *45*) for electron spectroscopy and with scattering functions derived for neutron and x-ray spectroscopies (*46*), implies that only phonon modes with a component along the direction of imparted momentum transfer will contribute to the phonon spectrum.

As the loss function is not well defined at **q** = 0 (the cross section diverges because of a ~1/**q**^2^ dependence), a **q** value of 0.01 Å^−1^ was used for the corresponding modeled spectra. For simplicity, the simulated **q** = 0.01 Å^−1^ spectra in Fig. 1 (E and F) are referred to as **q** ~ 0 Å^−1^ in the text. In Fig. 3A, a modeled spectrum for $\left[7\bar{8}13\right]$ electron beam incidence is superimposed on the experimental aloof spectrum (distance, 55 nm). The modeled spectrum is normalized to the experimental LO peak height and comprises the summation of two spectra with **q** = 0.01 Å^−1^ along the Γ→$01\bar{1}3$ and $\Gamma \to 10\bar{12}$ directions (see Fig. 3, D and E).

## Supplementary Material

http://advances.sciencemag.org/cgi/content/full/4/6/eaar7495/DC1

## SUPPLEMENTARY MATERIALS

Supplementary material for this article is available at http://advances.sciencemag.org/cgi/content/full/4/6/eaar7495/DC1 fig. S1. Selected momentum-resolved experimental EEL spectra before background subtraction. fig. S2. Spatially resolved vibrational EELS of hBN for electron beam incidence parallel to the crystallographic *c* axis.
