# Supplementary material for: Nanoscale momentum-resolved vibrational spectroscopy
Source: Sci Adv. 2018 Jun 15;4(6):eaar7495. doi: 10.1126/sciadv.aar7495 (PMC6018998; doi:10.1126/sciadv.aar7495)
Supplement: http://advances.sciencemag.org/cgi/content/full/4/6/eaar7495/DC1 [file supp_4_6_eaar7495__index.html]

Science Advances | Science Advances

## Supplementary Materials

**This PDF file includes:**

- fig. S1. Selected momentum-resolved experimental EEL spectra before background subtraction.
- fig. S2. Spatially resolved vibrational EELS of hBN for electron beam incidence parallel to the crystallographic *c* axis.

Download PDF

**Files in this Data Supplement:**

- Adobe PDF - aar7495\_SM.pdf
