# Supplementary material for: Nanoscale momentum-resolved vibrational spectroscopy
Source: Sci Adv. 2018 Jun 15;4(6):eaar7495. doi: 10.1126/sciadv.aar7495 (PMC6018998; doi:10.1126/sciadv.aar7495)
Supplement: http://advances.sciencemag.org/cgi/content/full/4/6/eaar7495/DC1 [file aar7495_SM.pdf]

Science Advances

AAAS

advances.sciencemag.org/cgi/content/full/4/6/eaar7495/DC1

Supplementary Materials for

Nanoscale momentum-resolved vibrational spectroscopy

Fredrik S. Hage, Rebecca J. Nicholls, Jonathan R. Yates, Dougal G. McCulloch, Tracy C. Lovejoy, Niklas Dellby, Ondrej L. Krivanek, Keith Refson, Quentin M. Ramasse

Published 15 June 2018, *Sci. Adv.* **4**, eaar7495 (2018)
DOI: 10.1126/sciadv.aar7495

This PDF file includes:

- fig. S1. Selected momentum-resolved experimental EEL spectra before background subtraction.
- fig. S2. Spatially resolved vibrational EELS of hBN for electron beam incidence parallel to the crystallographic *c* axis.

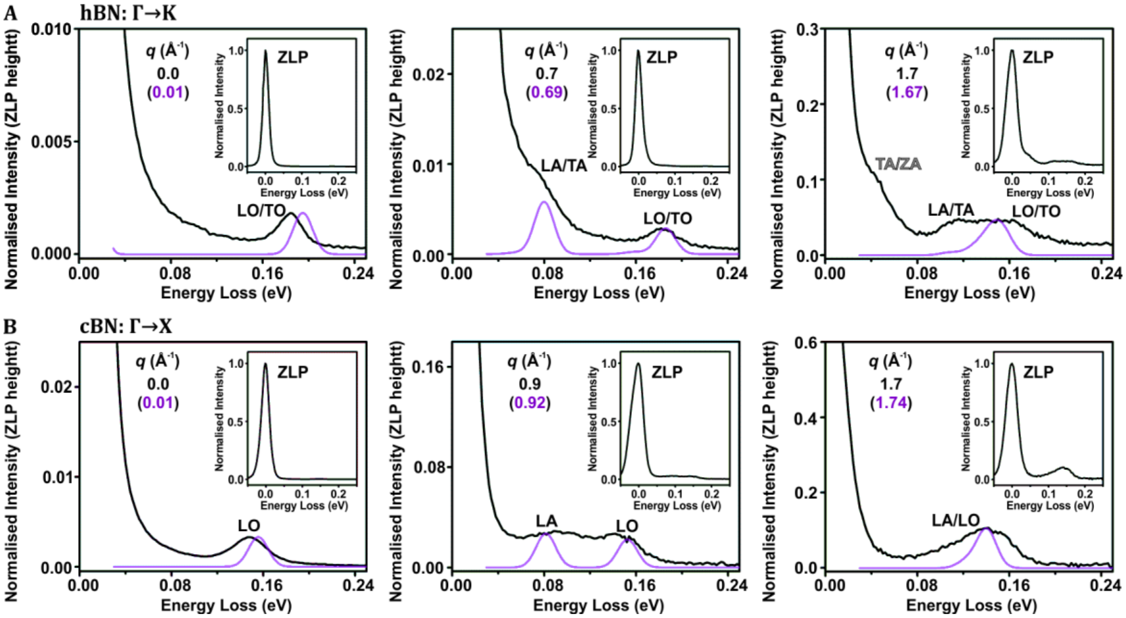

fig. S1. Selected momentum-resolved experimental EEL spectra before background subtraction. (**A**) The hBN spectra shown in Fig. 1E. (**B**) The cBN spectra shown in Fig. 1F. All experimental spectra are normalised to their respective ZLP heights. Superimposed simulated (in-plane polarisation only, purple) EEL spectra are scaled to the intensities of the phonon loss peaks of their respective experimental counterparts. The magnitude of displacement in momentum space considered for each spectrum is given in black (experimental spectra, corresponding to the centre of the displaced beam, estimated to one decimal place) and purple (modelled spectra).

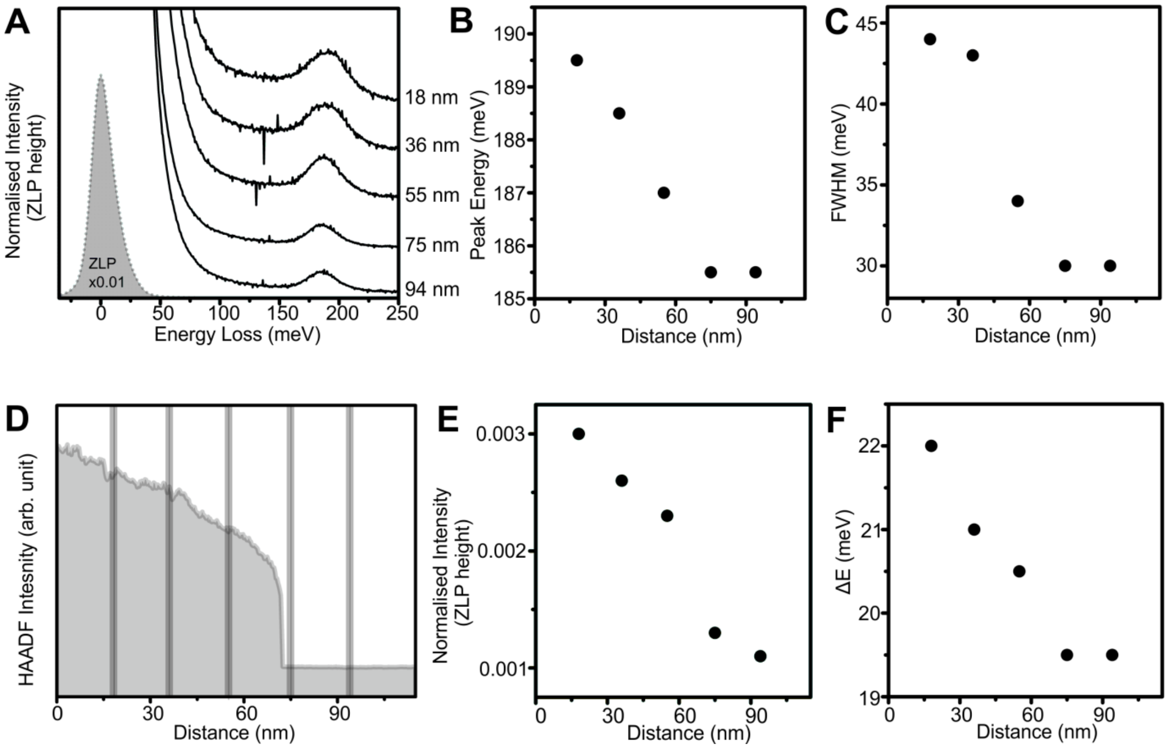

fig. S2. Spatially resolved vibrational EELS of hBN for electron beam incidence parallel to the crystallographic *c* axis. (**A**) Spatially resolved loss spectra acquired from a hBN flake in the regions indicated in the HAADF line profile in (**D**). The plots show LO/TO peak parameters: (**B**) peak energy, (**C**) FWHM and (**E**) relative intensity with respect to the ZLP height. (**F**) Shows the full-width at half-maximum (ΔE) of the ZLP for each spectrum.
